# Systematic review and meta-analysis of the prognostic value of CXCR2 in solid tumor patients

**DOI:** 10.18632/oncotarget.22285

**Published:** 2017-11-03

**Authors:** Yong Yang, Baoyang Luo, Yong An, Han Sun, Huihua Cai, Donglin Sun

**Affiliations:** ^1^ Department of Hepatobiliary Pancreatic Surgery, The Third Affiliated Hospital of Soochow University, Changzhou 213003, People’s Republic of China; ^2^ Department of Orthopedic Surgery, The Third Affiliated Hospital of Soochow University, Changzhou 213003, People’s Republic of China

**Keywords:** CXCR2, survival, solid tumor, biomarker, meta-analysis

## Abstract

CXC chemokine receptor-2 (CXCR2) expression is associated with the prognosis of multiple cancers. We performed a meta-analysis to determine the association between the CXCR2 expression in tumor tissue and patient prognosis. We compiled related literature from PubMed, Embase, and Web of Science (last updated July 31, 2017). A total of 4012 patients with solid tumors from 21 studies were included to evaluate the association between CXCR2 and overall survival, recurrence-free survival, or disease-free survival. High CXCR2 expression was significantly associated with poor overall survival (pooled HR = 1.82; 95% CI = 1.63–2.03; *P* < 0.001), recurrence-free survival (pooled HR = 1.40; 95% CI = 1.21–1.62; *P* < 0.001), and disease-free survival (pooled HR = 1.89; 95% CI = 1.05–3.40; *P =* 0.033), especially in patients with digestive system neoplasms. Thus high CXCR2 expression in tumor tissue appears predictive of a poor prognosis in patients with solid tumors. Further studies will be required to determine whether CXCR2 blockade has a favorable effect on the prognosis of patients with cancer.

## INTRODUCTION

Cancer is a leading cause of mortality worldwide according to the latest statistics from the American Cancer Society. In 2016, there were approximately 1.7 million new cancer cases and 0.6 million cancer-related deaths in the United States [1]. Many solid tumors lack specific tumor biomarkers and effective treatment options. Novel therapeutic approaches and tumor biomarkers with high specificity and sensitivity are necessary for the diagnosis and outcome evaluation of cancer patients.

Chemokines are a group of small molecular proteins that bind to corresponding cell-surface receptors and participate in various immunological, physiological, and pathological processes. There are nearly 50 human genes that encode chemokine ligands, and more than 20 corresponding chemokine receptors. Chemokines are further divided into four classes according to structural differences: CXC, CC, CX3C, and C [2]. Emerging evidence indicates that CXC chemokines and their receptors contribute to tumor-related processes including tumor cell proliferation, invasion, metastasis, and angiogenesis [3]. CXC chemokines are further divided into ELR+ and ELR- subtypes according to the presence or absence of an ELR (Glu-Leu-Arg) tripeptide motif at the NH2 terminus.

Multiple receptors have been identified as CXCR1-CXCR7 [4]. CXC chemokine receptor-2 (CXCR2) is a primary receptor of the CXC superfamily and has a high affinity for chemokines [5]. This seven-transmembrane G protein-coupled receptor is expressed on cell membranes of leukocytes, endothelial cells, and tumor cells. Chemokine-receptor binding can promote tumor cell proliferation and invasion as well as tumor tissue angiogenesis, regulate the association between tumor cell and extracellular matrix, and mediate immune cell infiltration and drug resistance [4, 6–10]. CXCR2 has been associated with to the biological behavior of tumors in colon cancer [11], oral squamous cell carcinoma [12], pancreatic cancer [13], and hepatocellular carcinoma [14].

Several studies have shown that CXCR2 overexpression was associated with poor survival in gastric cancer [15], hepatocellular cancer [16], and renal cell cancer [17]. Other studies revealed had no obvious association of CXCR2 with the outcomes of esophageal cancer [18], pancreatic ductal cancer [19], or ovarian cancer [7]. We conducted a systematic review and meta-analysis to clarify the prognostic effect of elevated CXCR2 levels in solid tumors.

## RESULTS

### Study characteristics

**Figure 1:**
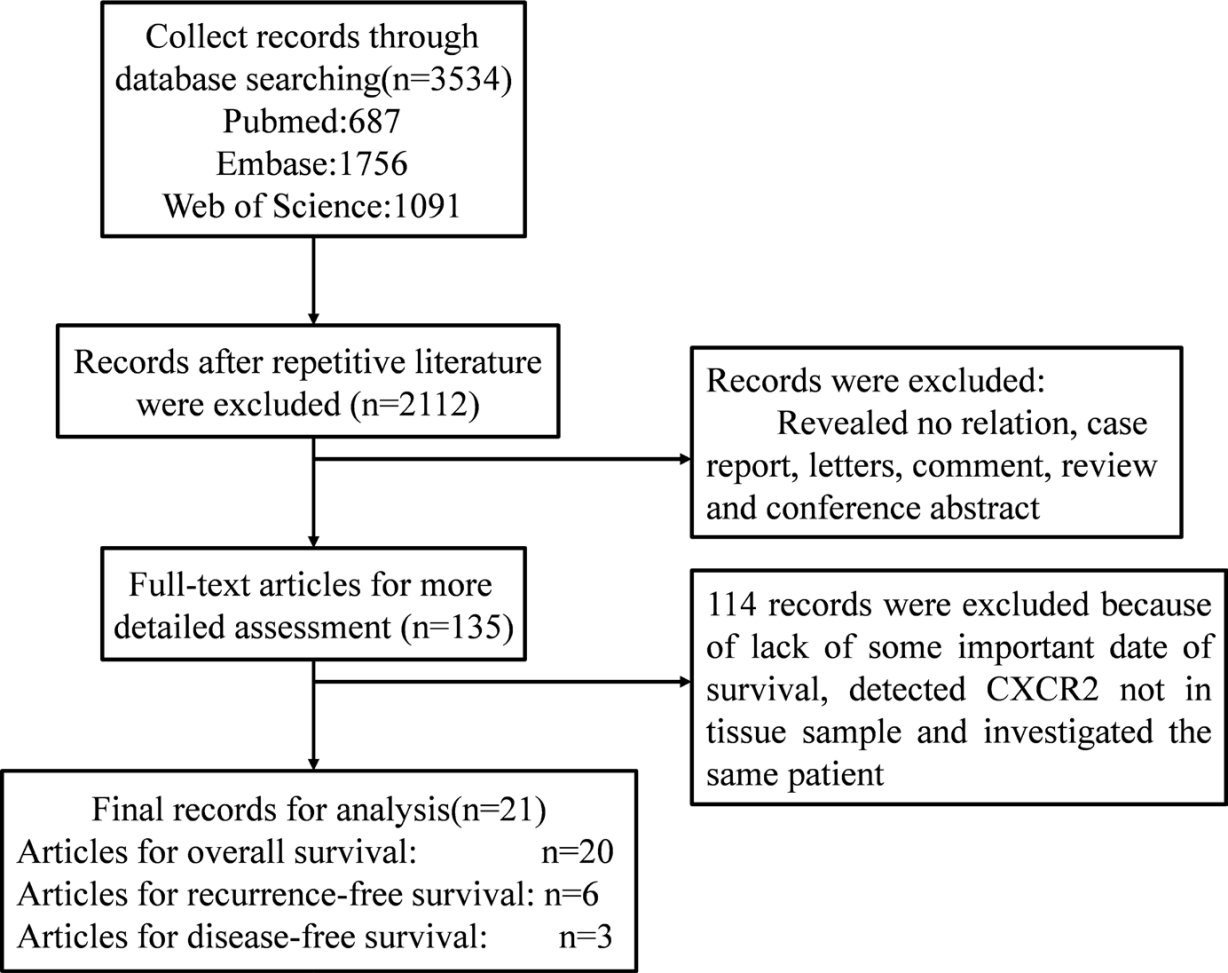
Scheme of the study selection process

According to the described searching strategy, 3534 records were initially collected. After initial screening of titles, abstracts, and full text of each publication to exclude non-conforming articles, 135 records were obtained. Then 114 full-text articles were further excluded due to lack of survival data, CXCR2 detected in a non-tissue sample, or investigation of the same patient. Finally, 21 articles were enrolled in this meta-analysis. Figure 1 describes the detailed study selection process.

Primary information of the included studies is shown in Table 1. A total of 4012 patients from USA, China, Japan, Iran, and Greece were diagnosed with hepatocellular carcinoma, esophageal cancer, gastric cancer, non-small cell lung cancer, laryngeal squamous cell carcinoma, ovarian cancer, renal cell carcinoma, pancreatic ductal adenocarcinoma, colorectal cancer, or astrocytic tumors. Among these studies, 3894 patients from 20 studies were evaluated by overall survival (OS), 1450 patients from 6 studies were evaluated by recurrence-free survival (RFS), and 377 patients from 3 studies were evaluated by disease-free survival (DFS). The 2411 patients from 13 studies were diagnosed with cancers of the digestive system. All studies were published in 2010 or later and assessed CXCR2 expression in tumor tissue by immunohistochemistry. All tumor tissues were derived from surgical specimens. Asian subjects comprised 15 studies, and 6 studies were on Caucasians. Hazard ratios (HRs) and 95% confidence intervals (95% CIs) were reported directly in 13 studies and estimated indirectly from the other 8 studies. The co-variables of the studies controlled for by multivariable Cox regression are shown in Table 2 and the cut-off values in these studies varied.

**Table 1:** Characteristics of studies included in the meta-analysis

Study ID	Year	Country	Cancer	Number	High-expression n (%)	Stage	Other treatments	Cut-off	Outcome	HR	Multivariate or Univariate analysis	Follow-up (months)
Gold [39]	2014	USA	NSCLC	370	186 (50.3%)	I–IIIA	Before surgery (128)	Median	OS/RFS	R	Multivariate	Median 64
Saintigny [40]	2013	USA	NSCLC	262	121 (46.2%)	I–II	no	H-score > 20	OS/RFS	R	Multivariate	Median 64
Han [41]	2012	China	LSCC	109	73 (67.0%)	I–IV	no	IRS ≥ 3	OS	SC	Univariate	12–120
Li [16]	2015	China	HCC	259	129 (49.8%)	I–IV	no	Median	OS/RFS	R	Multivariate	Over 60
Zhou [35]	2015	China	HCC	452	226 (50.0%)	I–IV	Unknown	Median	OS	R	Multivariate	Over 60
Yang [7]	2010	USA	OC	240	90 (37.5%)	Early-Late	no	Median	OS/DFS	R	Multivariate	24–240
An [17]	2015	China	RCC	375	123 (32.8%)	T1–4	no	H-score > 190	OS/RFS	R	Multivariate	105 (12–120)
Rezakhaniha [42]	2016	Iran	RCC	45	36 (80%)	I–IV	Unknown	≥ 30% of cell stained	OS	SC	Univariate	Over 60
Sui [43]	2013	China	EC	95	55 (57.9%)	I–III	Unknown	IRS ≥ 8	OS	R	Multivariate	Over 60
Wu [44]	2016	China	EC	156	74 (47.4%)	I–III	no	>30% of cell stained	OS	SC	Univariate	12–84
Nishi [18]	2015	Japan	EC	82	33 (40.2%)	I–III	no	+/-	OS/RFS	SC	Univariate	Over 60
Xiang [33]	2017	China	GC	115	67 (58.3%)	I–IV	no	IRS > 6.7	OS	SC	Univariate	Median 62.9
Kasashima [45]	2017	Japan	GC	270	113 (41.9%)	I–IV	Unknown	IRS ≥ 4	OS	R	Multivariate	Up to 60
Cheng [46]	2010	China	GC	116	61 (52.6%)	I–IV	Unknown	H-score > 90	OS	SC	Univariate	Mean 60.2
Wang [15]	2016	China	GC	357	200 (56.0%)	I–IV	no	H-score >200	OS	R	Multivariate	Median 41
Yang [47]	2015	China	GC	112	64 (57.14%)	I–IV	no	> 30% of cell stained	OS	R	Multivariate	Over 60
Korkolopoulou [48]	2011	Greece	AT	82	34 (41.5%)	II–IV	Unknown	+/-	OS	R	Multivariate	6–104
Maeda [19]	2017	Japan	PDAC	102	63 (61.8%)	I–IV	After surgery	≥ 50% of cell stained	OS/RFS	SC	Univariate	3–96
Wang [49]	2014	China	PDAC	161	87 (54.0%)	I–IV	After surgery	H-score > 140	OS	R	Multivariate	Over 60
Zhao [50]	2017	China	CRC	134	82 (61.2)	I–IV	no	IRS ≥ 6	OS/DFS	R	Multivariate	48–60
Stofas [51]	2014	Greece	RCC	118	56 (47.5%)	I–IV	no	H-score > 80	DFS	SC	Univariate	1.77–117.43

NSCLC: non-small cell lung cancer; LSCC: laryngeal squamous cell carcinoma; HCC: hepatocellular carcinoma; OC: Ovarian Cancer; RCC: renal cell carcinoma; EC: esophageal cancer; GC: gastric cancer; AT: Astrocytic Tumors; PDAC: Pancreatic Ductal Adenocarcinoma; CRC: Colorectal cancer IHC: immunohistochemistry; OS: overall survival; DFS: disease-free survival; RFS: recurrence-free survival; R: report; SC: survival curve; IRS: immunoreactivity score.

**Table 2:** Co-variables controlled for by studies using multivariable Cox regression

Study ID	Outcome	Co-variables
Gold [39]	OS	Age, Stage, c-pAMPK, c-pmTOR, c-EPCAM, n-FEN1
	RFS	Age, Stage, c-pAMPK, c-pmTOR, c-EPCAM, c-IGF-1R, m-Insulin receptor, m-CASK
Saintigny [40]	OS	Age, Gender, Stage
	RFS	Age,, Stage
Li [16]	OS/RFS	Age, Gender, HBsAg, Cirrhosis, ALT, AST, AFP, Tumor size, Tumor differentiation, Vascular invasion,
		Tumor multiplicity, TNM stage, BCLC stage
Zhou [35]	OS	AFP, GGT, Liver cirrhosis, Tumor size, Microvascular invasion, Tumor encapsulation, Tumor differentiation
Yang [7]	OS/DFS	Age, Stage, Family history, Subtype, Clinical response, Ascities
An [17]	OS/RFS	Tumor size, Stage, Fuhrman grade, Necrosis, ECOG-PS
Sui [43]	OS	Age, Gender, TNM stage, Lymph node metastasis, Tumor dimension
Kasashima [45]	OS	CXCL1, CXCL1 and CXCR2 both, Macroscopic type, Histological type, T invasion, Lymph node metastasis,
		Lymphatic invasion, Venous invasion, Hepatic metastasis, Peritoneal metastasis, Peritoneal cytology
Wang [15]	OS	T stage, Lymph node metastasis, Distant metastasis, Lauren classification
Yang [47]	OS	Age, IL-22BP expression, TNM stage, Depth of invasion, Lymph node metastasis
Korkolopoulou [48]	OS	Surgery, Radiotherapy, Histological grade
Wang [49]	OS	Age, Gender, TNM stage, Lymph node metastasis, Tumor size, Tumor differentiation, Vascular invasion,
		Tumor location, Perineural invasion, Surrounding tissue invasion
Zhao [50]	OS/DFS	Dukes stage

c: cytoplasmic, m: membrane, n: nuclear, AFP alpha-fetoprotein, ALT alanine aminotransferase, AST aspartate aminotransferase, BCLC Barcelona Clinic Liver Cancer, HBsAg hepatitis B surface antigen, TNM tumor-nodes-metastasis, GGT, gamma glutamyl transferase, ECOG-PS, Eastern Cooperative Oncology Group performance status, IL-22BP, interleukin-22 receptor 2.

### Quality assessment

We used the Quality in Prognostic Studies (QUIPS) tool to assess the quality of the 21 eligible studies included in our meta-analysis [20] (Supplementary Table 1). A moderate risk of bias for domain 1 (Study Participation) was shown in 4 original studies and was primarily due to small participation cohorts. Moderate bias in domain 2 (Study Attrition) was indicated for 1 original study due to missing data from participants without a follow-up. Moderate bias in domain 3 (Prognostic Factor Measurement) was shown in 6 original studies due to a lack of definite cutoff values. All original studies provided clear outcome definitions and had a low risk of bias for domain 4 (Outcome Measurement). Moderate bias in domain 5 (Study Confounding) was shown in 6 original studies that lacked detailed descriptions of treatment beyond surgery. Moreover, 8 original studies conducted only univariate Cox analysis and had a moderate risk of bias for domain 6 (Statistical Analysis and Reporting).

### CXCR2 and OS

**Figure 2:**
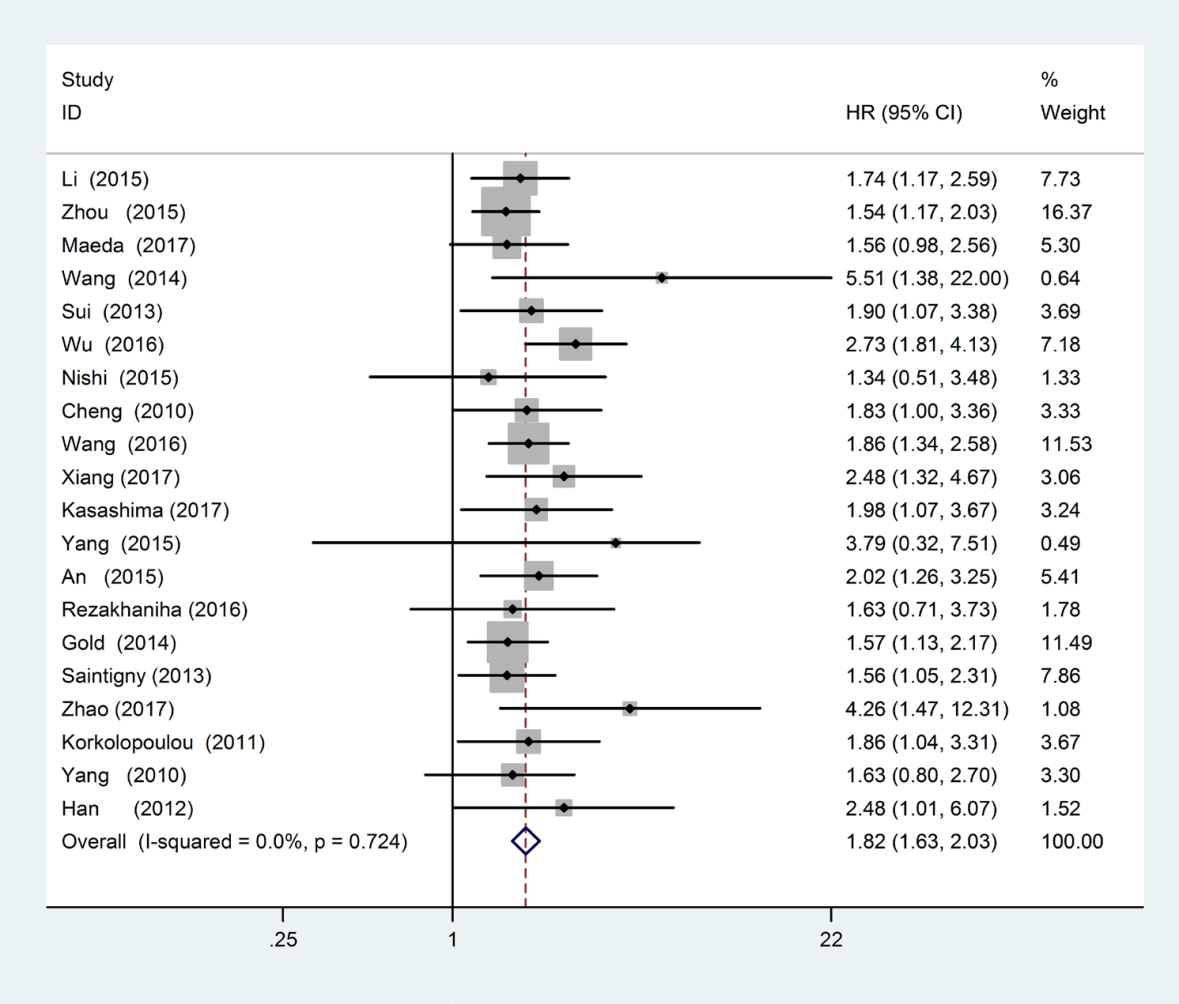
Forest plots of studies evaluating hazard ratios of high CXCR2 expression in solid tumors for overall survival (OS)

The 20 studies including 3894 patients provided correlative data for OS analysis. We used the fixed-effects model to calculate the pooled HR and its 95% CI due to no obvious heterogeneity (*P* = 0.724, I^2^ = 0.0%). The pooled HR was 1.82 (95% CI = 1.63–2.03, *P* < 0.001), and indicated that increased CXCR2 expression might predict poor OS in solid tumor patients (Figure 2).

**Figure 3:**
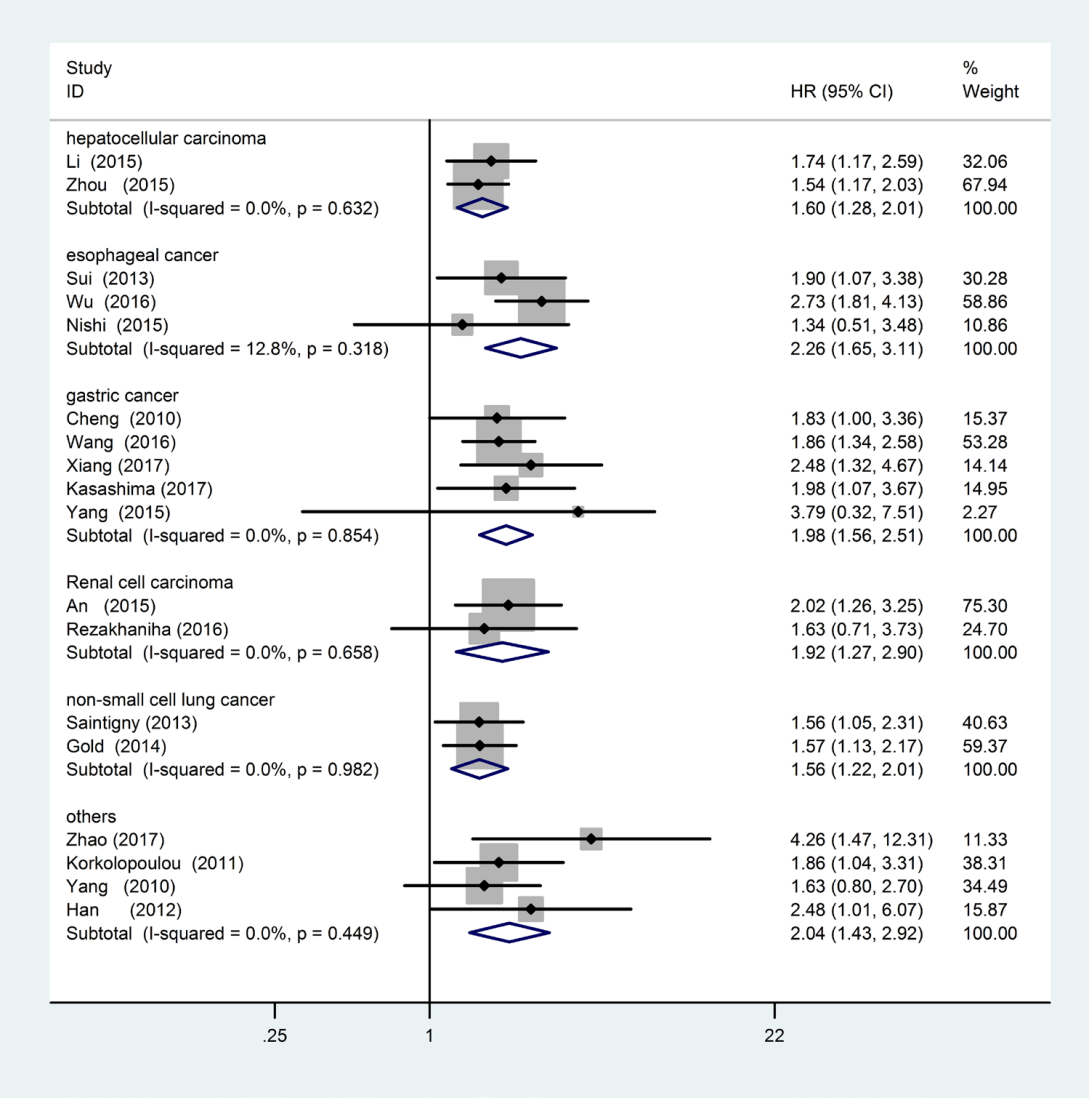
Forest plot of tumor type subgroup analysis

Subgroup analyses were carried out according to tumor type, tumor source, analysis type, and ethnicity. Heterogeneity was found in the pancreatic ductal adenocarcinoma group (*P* = 0.091, I^2^=65%), so we used a random-effect model to calculate the pooled HR and its 95% CI. The CXCR2 expression had no significant association with OS in pancreatic ductal adenocarcinoma (pooled HR = 2.47; 95% CI = 0.75–8.10; *P* = 0.137). There was no obvious heterogeneity in other subgroups, so we used the fixed-effects model to calculate the pooled HR and its 95% CI. These results are shown in Table 3. According to tumor type, CXCR2 overexpression predicted poor OS for patients with hepatocellular carcinoma (HR = 1.60; 95% CI = 1.28–2.01; *P* < 0.001), esophageal cancer (HR = 2.26; 95% CI = 1.65–3.11; *P* < 0.001), gastric cancer (HR = 1.98; 95% CI = 1.56–2.51; *P* < 0.001), renal cell carcinoma (HR = 1.92; 95% CI = 1.27–2.90; *P* = 0.002), non-small cell lung cancer (HR = 1.56; 95% CI = 1.22–2.01; *P* < 0.001) and others tumor type (HR = 2.04; 95% CI = 1.43–2.92; *P* < 0.001) (Figure 3).

**Table 3:** Pooled OS HRs according to subgroup analyses

Subgroup	Study	No. of patients	Fixed-effects model		Heterogeneity	
			HR (95%CI)	*P* value	I^2^ (%)	*P*
Overall survival		3894	1.82 (1.63–2.03)	< 0.001	0	0.724
Tumor type						
HCC	[16, 35]	711	1.60 (1.28–2.01)	< 0.001	0	0.632
EC	[18, 43, 44]	333	2.26 (1.65–3.11)	< 0.001	12.8	0.318
GC	[15, 33, 45–47]	970	1.98 (1.56–2.51)	< 0.001	0	0.854
RCC	[17, 42]	420	1.92 (1.27–2.90)	0.002	0	0.658
NLCC	[39, 40]	632	1.56 (1.22–2.01)	< 0.001	0	0.982
Others	[7, 41, 48, 50]	565	2.04 (1.43–2.92)	< 0.001	0	0.449
Tumor source						
DSN	[15, 16, 18, 19, 33, 35, 43–47, 49, 50]	2411	1.88 (1.64–2.16)	< 0.001	3.9	0.407
Others	[7, 17, 39–42, 48]	1483	1.70 (1.41–2.05)	< 0.001	0	0.942
Analysis type						
multivariate	[7, 15–17, 35, 39, 40, 43, 45, 47–50]	3169	1.75 (1.54–1.99)	< 0.001	0	0.732
univariate	[18, 19, 33,41, 42, 44, 46]	725	2.06 (1.64–2.59)	< 0.001	0	0.570
Ethnicity						
Asian	[15–19, 33, 35, 41, 43–47, 49, 50]	2895	1.91 (1.67–2.17)	< 0.001	0	0.534
Caucasian	[7, 39, 40, 42, 48]	999	1.61 (1.31–1.99)	< 0.001	0	0.990

HR: hazard ratio; CI: confidence interval; DSN: digestive system neoplasms; HCC: hepatocellular carcinoma; EC: esophageal cancer; GC: gastric cancer; RCC: renal cell carcinoma, NSCLC: non-small cell lung cancer.

**Figure 4:**
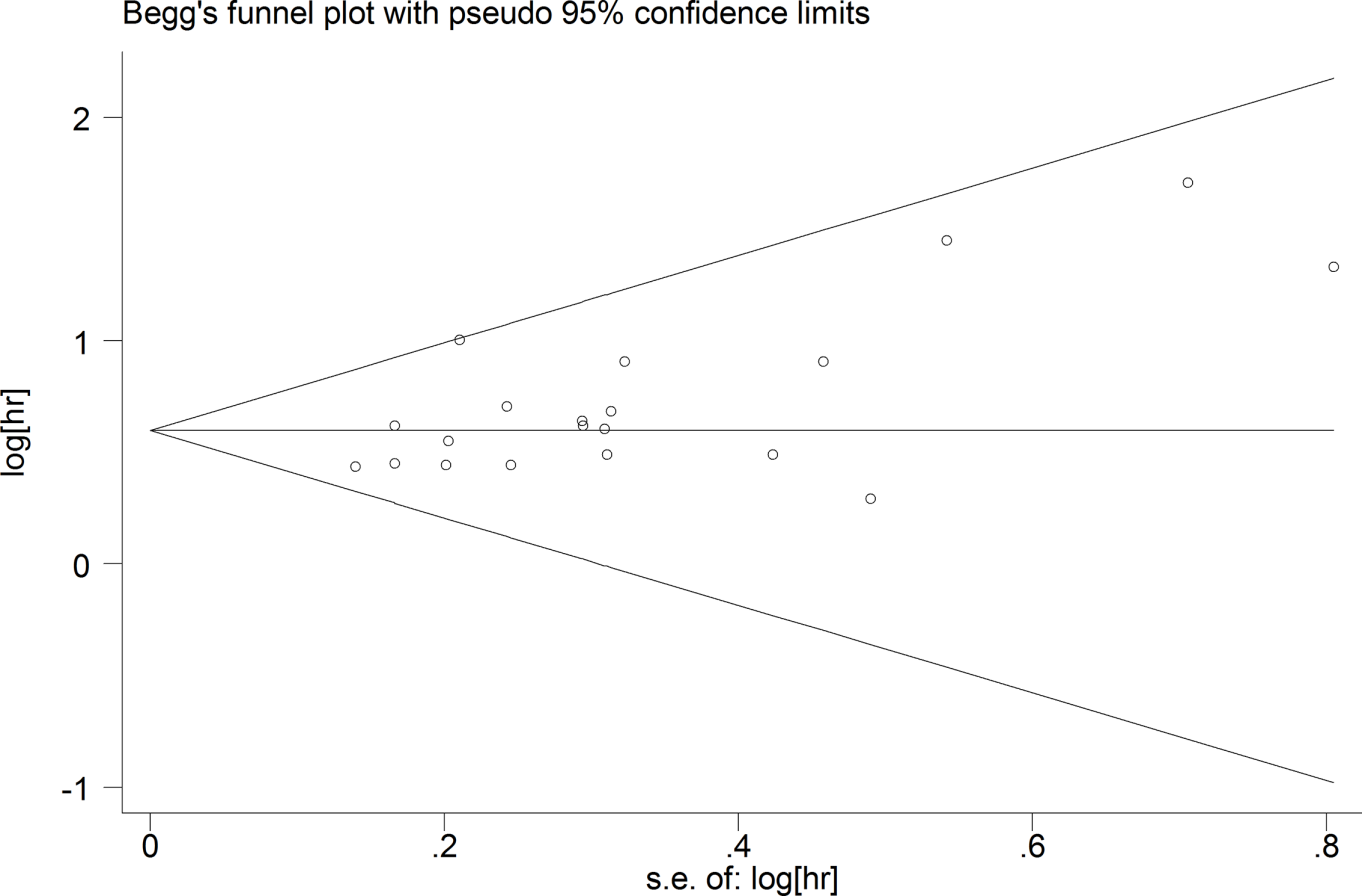
Funnel plots of publication biases of association between CXCR2 expression and overall survival (OS) in solid cancer patients

Funnel plots, Egger’s, and Begg’s tests were used to evaluate the publication bias of all included studies. There was obvious publication bias because of the *P*-value of the Egger’s regression intercept was 0.012 (Figure 4). We used the “Trim and Fill” method to adjust for publication bias under the fixed-effects model, and the corrected multivariable-adjusted pooled HR for OS was 1.78 (95% CI = 1.60–2.00).

### CXCR2 and RFS

**Figure 5:**
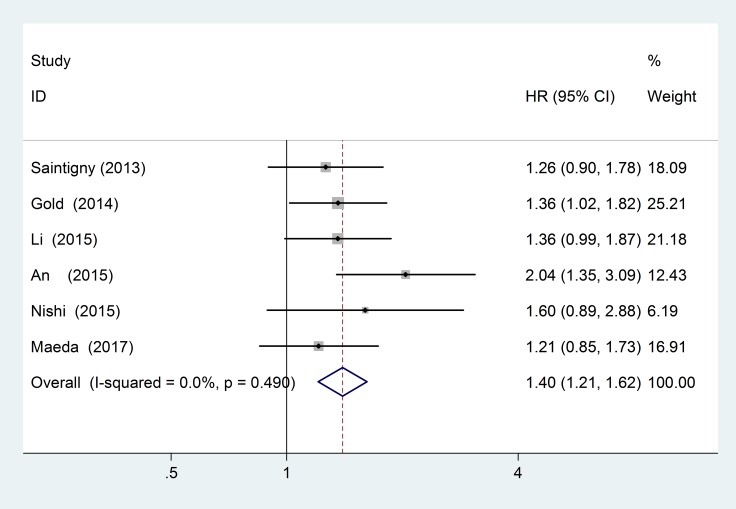
Forest plots of studies evaluating hazard ratios (HR) of high CXCR2 expression in solid tumors for recurrence-free survival analysis (RFS)

In this meta-analysis, 6 studies with a total of 1450 patients provided suitable data for RFS analysis. No obvious statistical heterogeneity (I^2^ = 0.0%; *P* = 0.490) was found, so we used a fixed-effects model to pool the HR. High CXCR2 expression was significantly associated with poor RFS (pooled HR = 1.40; 95% CI = 1.21–1.62; *P* < 0.001) (Figure 5). The subgroup analyses results are shown in Table 4.

**Table 4:** Pooled RFS HR according to subgroup analyses

Subgroup	Study	No. of patients	Fixed-effects model		Heterogeneity	
			HR(95% CI)	*P* value	I^2^ (%)	*P*
RFS		1450	1.40 (1.21–1.62)	< 0.001	0.0%	0.490
Ethnicity						
Asian	[16–19]	818	1.46 (1.20–1.77)	< 0.001	22.3%	0.277
Caucasian	[39, 40]	632	1.32 (1.06–1.65)	0.015	0.0%	0.747
Tumor source						
DSN	[16, 18, 19]	443	1.33 (1.07–1.66)	0.011	0.0%	0.716
Others	[17, 39, 40]	1007	1.45 (1.19–1.77)	< 0.001	41.3%	0.182
Analysis type						
multivariate	[16, 17, 39, 40]	1266	1.43 (1.21–1.68)	< 0.001	15.1%	0.317
univariate	[18, 19]	184	1.30 (0.96–1.77)	0.087	0.0%	0.425

RFS: recurrence-free survival; HR: hazard ratio; CI: confidence interval; DSN: digestive system neoplasms.

### CXCR2 and DFS

**Figure 6:**
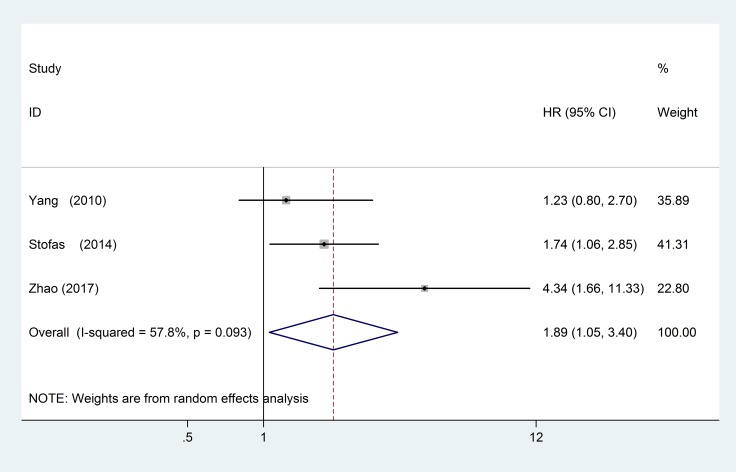
Forest plots of studies evaluating hazard ratios (HR) of high CXCR2 expression in solid tumors for disease-free survival analysis (DFS)

Correlative data for DFS analysis was provided in 3 studies including 377 patients. The studies that evaluated DFS had statistical heterogeneity (I^2^ = 57.8%, *P* = 0.093), so we used a random-effects model to pool the HR. High CXCR2 expression was significantly associated with poor DFS (pooled HR = 1.89; 95% CI = 1.05–3.40; *P* = 0.033) (Figure 6).

## DISCUSSION

CXC chemokines are a group of small molecule proteins with similar structure and function that are secreted by various cells *in vivo*. They primarily induce leukocyte accumulation in lesions and sites of inflammation [21]. Most ELR+ CXC chemokines, including CXCL1–3 and CXCL5–8, increase the tumor tissue penetration of immunosuppressive cells, reduce apoptosis, and promote angiogenesis as well as tumor cell proliferation, migration, and invasion by activating the CXCR2 receptor [6–10, 22]. Therefore, CXCR2 might play a critical role in cancer progression.

Tumor progression depends on adequate blood supply, and there is evidence that CXCR2 is essential for tumor angiogenesis. Previous reports have demonstrated that the blockade of CXCR2 can significantly inhibit the formation of microvessels in tumor tissue [23–25] The activation of CXCR2 promotes tumor angiogenesis through the following mechanisms: (1) increase the migration and tube formation of human umbilical vein endothelial cells (HUVECs) [26]; (2) promote vasculogenic mimicry (VM) in tumors [27], which is a non-classical mechanism whereby cancer cells, rather than endothelial cells, form blood vessels; (3) increase the expression of vascular endothelial growth factor (VEGF) in tumor tissues [28]; (4) regulate migratory and angiogenic activities of endothelial progenitor cells (EPCs) and induce tumor tissue angiogenesis [29]; (5) induce tumor-infiltrating myeloid cells of tumor tissues, which are known to support blood vessel development in solid tumor growth [30].

Blockade of the CXCR2 signaling pathway can significantly enhance the effect of chemotherapy in colon cancer patients [31] and inhibit the proliferation and invasion of chemotherapy-resistant breast cancer cells [32]. The activation of this signaling pathway can also promote the invasion and metastasis of gastric cancer cells [33] as well as the progression of bladder cancer by inducing the penetration of myeloid-derived inhibitory cells [34]. Activation of CXCR2 mainly induced the PI3K/Akt/GSK-3β/Snail signaling pathway and the subsequent epithelial- mesenchymal transition (EMT) to promote tumor cell invasion and metastasis [35]. CXCR2 also promoted tumor cell proliferation by modulating cell cycle regulatory proteins and inhibited cellular apoptosis by suppressing phosphorylated p53 and PUMA [7]. Moreover, CXCR2 can induce the accumulation of inflammatory cells to promote a local inflammatory response [36], which is closely related to the development of tumors [37]. Therefore, CXCR2 overexpression in tumor microenvironment could be associated with poor prognosis of cancer patients.

We provided strong evidence that CXCR2 overexpression in tumor tissue was an independent predictor of poor OS, RFS, and DFS in most cancers regardless of the ethnic background of patients in this meta-analysis. The subgroup analyses revealed that high CXCR2 expression was associated with poor prognosis of most digestive system cancers, including hepatocellular carcinoma, gastric cancer, and esophageal cancer. The results tended to be inversely related to the prognosis of patients with pancreatic ductal adenocarcinoma but were not statistically significant. However, there was distinct heterogeneity in the pancreatic ductal adenocarcinoma subgroup, and the corresponding sample size was small. Therefore, larger samples are needed to further evaluate the association between CXCR2 expression and the prognosis of patients with pancreatic cancer.

This was the first meta-analysis to demonstrate that high CXCR2 expression was significantly associated with poor prognosis of most solid tumors. There were limitations to our analysis. This study included 20 related studies. The cut-off value of each study was different so there was a lack of uniform cut-off values when CXCR2 was used as a predictive biomarker of cancer prognosis. A unified cut-off value should be further defined. Some HRs were indirectly calculated from data extracted from the survival curves, which could result in small statistical errors. The different analysis modes, tumor types, follow-up times, and sample sources may have also lead to a statistical bias and affected our results.

Our results were the first to suggest that CXCR2 overexpression was associated with poor survival of patients with most cancer types. However, there was no clear evidence that CXCR2 was associated with the prognosis of pancreatic cancer patients. We demonstrated that CXCR2 might be a prognostic biomarker of some cancers. In addition, the blockade of CXCR2 receptors might contribute to novel cancer treatments. However, there were limitations in our study, and the relevant results require further investigation. Additional high-quality clinical research data and larger sample sizes are needed to characterize the role of CXCR2 expression in cancer further.

## MATERIALS AND METHODS

The meta-analysis was reported according to the Systematic Reviews and Meta-Analyses (PRISMA) statement [38] (Supplementary Table 2 ).

### Search strategy

Literature published before July 31, 2017, was searched in the PubMed, Embase, and Web of Science databases. The search keywords were “CXCR2 or CXC chemokine receptor-2” (all fields) AND “cancer or carcinoma or tumor or neoplasm” (all fields). There were no other limitations when searching the databases. The reference lists of relative articles were screened manually to avoid deviations in the search process. Two investigators (Y. Yong and L. Baoyang) independently conducted the study selection.

### Inclusion and exclusion criteria

The literature included in this meta-analysis met the following criteria: (1) study subjects were patients with any type of solid tumor; (2) CXCR2 expression was measured in cancer tissue; (3) the association between survival outcome and CXCR2 expression was investigated; (4) the HR and 95% CI was provided directly or was extracted from the survival curves. Articles were excluded according to the following criteria: (1) studies of cell lines, animals, or non-solid tumors; (2) reviews, letters and case reports; (3) articles without original data or the prognostic data beyond CXCR2 alone; (4) lack of survival outcome or related data for the estimation of HR and 95% CI.

### Data extraction and quality assessment

The relevant information was collected by two independent searches of all incorporated studies and included: first author, publication year, country, tumor type, sample number, tumor stage, detection method, cut-off value, follow-up period, and HR as well as the corresponding 95% CI for OS, RFS or DFS. We preferentially used the multivariate analysis result if the study reported included both univariate and multivariate analysis. The QUIPS tool [20] was used to assess the bias risk of each study. Risk of bias was graded as high, moderate, or low.

### Statistical analysis

High and low expression of CXCR2 was defined according to the cut-off values specified in the studies. We calculated the pooled data by using the HRs and their 95% CIs from each study. *P* < 0.05 indicated that CXCR2 expression was related to the prognosis of cancer patients. We evaluated the heterogeneity by the *Q* test and I^2^ statistic. We used a random-effect model when the data were heterogeneous (*P* ≤ 0.05 or I^2^ ≥ 50%). The fixed-effect model was used when the data indicated no obvious heterogeneity (*P* > 0.05 or I^2^ < 50%). The source of heterogeneity was identified through sensitivity and subgroup analyses. Meta-regression was also used to determine the factors contributing to the heterogeneities of some results. The Funnel plot and Egger test were used to analyze publication bias. All analyses were performed with Stata 12.0 software (Stata Corporation, College Station, TX, USA)

## SUPPLEMENTARY MATERIALS TABLES




