# Supplementary material for: Systematic review and meta-analysis of the prognostic value of CXCR2 in solid tumor patients
Source: Oncotarget. 2017 Nov 3;8(65):109740–51. doi: 10.18632/oncotarget.22285 (PMC5752557; doi:10.18632/oncotarget.22285)
Supplement: Supplementary file 1 [file oncotarget-08-109740-s001.pdf]

Systematic review and meta-analysis of the prognostic value of CXCR2 in solid tumor patients

SUPPLEMENTARY MATERIALS

Supplementary Table 1: Assessment of the included studies for risk of bias using the "Quality Assessment in Prognostic studies"(QUIPS) tool

Study ID	Risk of bias					
	Study Participation	Study Attrition	Prognostic Factor Measurement	Outcome measurement	Study Confounding	Statistical Analysis and report
Gold [39]	Low bias	Low bias	Moderate bias	Low bias	Low bias	Low bias
Saintigny [40]	Low bias	Low bias	Low bias	Low bias	Low bias	Low bias
Han [41]	Low bias	Low bias	Low bias	Low bias	Low bias	Low bias
Li [16]	Low bias	Low bias	Moderate bias	Low bias	Low bias	Low bias
Zhou [35]	Low bias	Low bias	Moderate bias	Low bias	Moderate bias	Low bias
Yang [7]	Low bias	Low bias	Moderate bias	Low bias	Low bias	Low bias
An [17]	Low bias	Low bias	Low bias	Low bias	Low bias	Low bias
Rezakhaniha [42]	Moderate bias	Low bias	Low bias	Low bias	Moderate bias	Moderate bias
Sui [43]	Moderate bias	Low bias	Low bias	Low bias	Moderate bias	Low bias
Wu [44]	Low bias	Low bias	Low bias	Low bias	Low bias	Moderate bias
Nishi [18]	Moderate bias	Low bias	Moderate bias	Low bias	Low bias	Moderate bias
Xiang [33]	Low bias	Low bias	Low bias	Low bias	Low bias	Moderate bias
Kasashima [45]	Low bias	Low bias	Low bias	Low bias	Moderate bias	Moderate bias
Cheng [46]	Low bias	Low bias	Low bias	Low bias	Moderate bias	Moderate bias
Wang [15]	Low bias	Low bias	Low bias	Low bias	Low bias	Low bias
Yang [47]	Low bias	Low bias	Low bias	Low bias	Low bias	Low bias
Korkolopoulou [48]	Moderate bias	Moderate bias	Moderate bias	Low bias	Moderate bias	Low bias
Maeda [19]	Low bias	Low bias	Low bias	Low bias	Low bias	Moderate bias
Wang [49]	Low bias	Low bias	Low bias	Low bias	Low bias	Low bias
Zhao [50]	Low bias	Low bias	Low bias	Low bias	Low bias	Low bias
Stofas [51]	Low bias	Low bias	Low bias	Low bias	Low bias	Moderate bias

Supplementary Table 2: PRISMA 2009 checklist. See Supplementary_Table_2
